# Prognostic Significance of the Tumour Microenvironment in Surgically Resected Canine Pulmonary Adenocarcinomas

**DOI:** 10.1111/vco.70075

**Published:** 2026-04-27

**Authors:** Masanao Ichimata, Yumiko Kagawa, Atsushi Toshima, Fukiko Matsuyama, Eri Fukazawa, Kei Harada, Ryuzo Katayama, Yuko Nakano, Tetsuya Kobayashi, Masaya Igase, Takuya Mizuno

**Affiliations:** ^1^ Laboratory of Molecular Diagnostics and Therapeutics, Joint Graduate School of Veterinary Medicine Yamaguchi University Yamaguchi Yamaguchi Japan; ^2^ Japan Small Animal Cancer Center, Public Interest Incorporated Foundation Japan Small Animal Medical Center Saitama Japan; ^3^ North Lab Sapporo Hokkaido Japan; ^4^ Public Interest Incorporated Foundation Japan Small Animal Medical Center Saitama Japan; ^5^ Division of Translational Research for One Medicine, Research Institute for Cell Design Medical Science Yamaguchi University Yamaguchi Yamaguchi Japan

**Keywords:** canine pulmonary adenocarcinoma, CD20+ B cells, CD8+ T cells, lymphoid aggregates, regulatory T cells, tertiary lymphoid structures, tumour‐infiltrating lymphocytes

## Abstract

The prognostic relevance of the tumour microenvironment in surgically resected canine pulmonary adenocarcinomas (cPACs) remains uncertain. This retrospective single‐centre cohort study evaluated associations between intratumoural immune features and postoperative outcomes. Dogs treated between 2005 and 2021 with histologically confirmed cPACs that had undergone surgical excision and had an evaluable tumour microenvironment were included. Immunohistochemistry for CD8, Foxp3, CD3 and CD20 was performed. CD8+ and Foxp3+ cell densities were quantified in five intratumoural hotspots of 0.237 mm^2^ each. Lymphoid aggregates (LAs) were defined as intratumoural aggregates located within tumour nests or intratumoural stroma and composed of at least 50 CD20+ B cells with intercellular spacing ≤ 10 μm and admixed CD3+ T cells. Primary outcomes were progression‐free interval (PFI) and overall survival time (OST). A total of 71 dogs were enrolled. LAs were identified in 49 dogs (69.0%). The median densities of CD8+, CD20+ and Foxp3+ cells were 130.0, 398.3 and 79.3 cells/mm^2^, respectively. Tumours with LAs showed higher CD20+ and Foxp3+ cell densities than tumours without LAs. Median PFI and OST were 754 days (95% CI, 375‐not reached) and 716 days (95% CI, 399–936), respectively. In multivariable analysis, tumour size classification, lymph node metastasis, surgical margin status and the presence of LAs were independently associated with shorter PFI. Age, lymph node metastasis and the CD8/Foxp3 ratio were independently associated with OST. These findings indicate that immunohistochemical features may provide additional prognostic information for postoperative risk stratification in cPACs and complement established clinicopathological factors.

## 1 Introduction

Canine primary pulmonary adenocarcinomas (cPACs) are rare malignancies that typically affect older dogs, and surgical lobectomy remains the standard local treatment [1]. Recent studies indicate a median overall survival of approximately 1–2 years after lobectomy for canine primary pulmonary carcinomas [2, 3, 4, 5, 6]. Clinicopathological factors influence postoperative outcomes, with the most consistent survival associations observed for the Canine Lung Carcinoma Stage Classification (CLCSC) tumour size category, surgical margin status and lymph node status [2, 3, 4, 5, 6]. Beyond these factors, molecular alterations have begun to inform prognosis. In particular, *human epidermal growth factor receptor* 2 (*HER2*) mutations, predominantly the V659E transmembrane variant, are associated with prolonged progression‐free interval (PFI) after resection, supporting a role for tumour genetics in postoperative risk stratification [7].

In human non–small cell lung cancer (NSCLC), the tumour microenvironment (TME) is recognised as an independent axis that complements clinicopathological and genomic factors in determining prognosis and therapeutic response [8, 9]. Higher densities of tumour‐infiltrating lymphocytes (TILs), particularly CD8+ cells but also CD3+ and CD4+ subsets, have been associated with improved survival in resected NSCLC in multiple meta‐analyses [8]. In parallel, intratumoural lymphoid structures, termed tertiary lymphoid structures (TLS), have been linked to favourable long‐term outcomes in early‐stage disease [10]. Moreover, TLS maturation and abundance correlate with major pathological response to neoadjuvant chemoimmunotherapy and with improved disease‐free survival in resectable NSCLC [11].

Recent studies are clarifying the role of the TME in the development and progression of malignant tumours in dogs, and the evidence base is steadily expanding [12, 13]. The prognostic significance of TILs has been documented in multiple veterinary studies, particularly in malignant neoplasms [12]. In cPACs, higher intratumoural regulatory T‐cell counts are associated with shorter overall survival time (OST) [14]. In contrast, evidence regarding organised immune architecture in canine malignant tumours remains limited. TLS‐consistent lymphoid aggregates (LAs) have been identified in canine cutaneous and subcutaneous sarcomas through immunohistochemistry and gene‐expression profiling [13], and standardised definitions with prognostic mapping across tumour types are still evolving. To date, no studies have evaluated the presence or prognostic significance of LAs in cPACs, and their impact on clinical outcomes remains unknown.

Therefore, we aimed to determine whether features of the TME in cPACs predict postoperative outcomes. Specifically, we evaluated whether the presence of LAs and the densities of CD8+ and Foxp3+ TILs are associated with PFI and OST following surgical resection, independent of established clinicopathological and molecular factors.

## 2 Material and Methods

### 2.1 Cell Line Validation Statement

Cell lines were not used in this study.

### 2.2 Study Design

This retrospective cohort study was performed at the Japan Small Animal Cancer Centre between April 2005 and December 2021. Dogs were enrolled if they underwent surgical excision of a pulmonary mass, were histologically diagnosed with PAC, and had TME data available for evaluation. Most cases overlapped with those from a prior study in cPACs [7], but the present study specifically focused on evaluating the prognostic significance of the TME. The TME was assessed for TIL density and for the presence of LAs. Medical records were reviewed to collect clinical, histopathologic, *HER2* mutation status, HER2 immunoreactivity scores, and survival data. The *HER2* mutation and the HER2 immunoreactivity score were determined as previously described [7, 15]. Tumour grades and histological patterns were reassessed by a pathologist (Y.K.) using the PPC grading system [16]. Data on the date and cause of death (classified as PAC‐related, other, unknown, or last confirmed survival date) were also collected. For incomplete follow‐up data, information was obtained from referring veterinarians or through telephone interviews with the owners. The presence of clinical signs was defined based on previously published criteria [2]. Perioperative death was defined as death within 14 days after surgery. Adjuvant chemotherapy was defined as cytotoxic chemotherapy administered within 4 weeks after surgery. Tumour staging and size classification followed the CLCSC system [2].

### 2.3 Immunohistochemistry Processing

Immunohistochemical (IHC) staining was performed to evaluate TIL subsets, including CD3+, CD8+, CD20+ and Foxp3+ cells, as previously described with minor modifications [17]. Briefly, formalin‐fixed, paraffin‐embedded tumour samples were sectioned at 4 μm thickness and mounted onto glass slides. Following deparaffinisation in xylene and rehydration through graded ethanol series to distilled water, antigen retrieval was carried out by heating the sections in 0.01 M citrate buffer (pH 6.0) at 121°C using an autoclave. Endogenous peroxidase activity was quenched by incubating sections in 3% hydrogen peroxide in methanol for 30 min at room temperature. Non‐specific protein binding was blocked with 5% skim milk and/or bovine serum albumin diluted in phosphate‐buffered saline (PBS) for 30 min. The slides were then incubated with primary antibodies specific for canine CD3 (clone CD3‐12, Abcam, Tokyo, Japan; 1:100 dilution), CD8 (rat anti‐canine CD8 monoclonal antibody, clone F3‐B2; 1:1000 dilution) [18], CD20 (rabbit anti‐CD20 polyclonal antibody, Thermo Fisher Scientific, Waltham, MA, USA; 1:100 dilution), or Foxp3 (mouse/rat anti‐FOXP3 monoclonal antibody, clone FJK‐16 s, Invitrogen, Carlsbad, CA, USA; 1:50 dilution) overnight at 4°C. After washing, sections were treated with a ready‐to‐use species‐appropriate secondary antibody reagent (Histofine, Nichirei Bioscience Inc., Tokyo, Japan) for 30 min at room temperature. Visualisation was achieved using a diaminobenzidine (DAB) chromogenic substrate (Nacalai Tesque Inc., Kyoto, Japan), and the slides were counterstained with Mayer's haematoxylin (FUJIFILM Wako, Osaka, Japan). Canine lymph node sections were used as positive controls for each marker to ensure the validity of the staining. For each staining run, isotype‐matched antibodies were used as negative controls to confirm specificity.

### 2.4 Immunohistochemistry Quantification and Analysis

IHC‐stained sections of PAC were scanned at ×200 using a whole‐slide scanner (Hamamatsu NanoZoomer; NDP.scan), and the images were digitised and analysed with NanoZoomer Digital Pathology software (NDP.view2; Hamamatsu, Japan).

LAs were defined, with reference to reports in NSCLC [19] and canine soft tissue sarcoma [13], as intratumoural aggregates located within tumour nests or intratumoural stroma, excluding aggregates restricted to the invasive margin or extratumoural regions, and composed of at least 50 CD20+ B cells with intercellular spacing ≤ 10 μm, admixed with variable proportions of CD3+ T cells.

For TIL density, we selected, for each case, five hotspots within tumour nests and intratumoural stroma in which CD8+, CD20+ and Foxp3+ cells were most abundant. Following prior reports, we excluded regions with necrosis, crush artefact, fibrosis, peribronchial areas and areas with very dense lymphocytic infiltration from TIL assessment [20, 21]. Hotspots were not selected randomly, but were chosen to provide a hotspot‐based assessment of intratumoural immune‐cell density in the setting of heterogeneous lymphocyte distribution within the tumour, with reference to previous IHC studies using manual counting of selected microscopic fields [22]. Each hotspot was defined as a circular region of 0.237 mm^2^ (radius, approximately 274 μm). Two manual counts were performed at 400× magnification, and the counts were converted to cell density (cells/mm^2^) for analysis. Lymphoid aggregates and TIL densities in cPACs were assessed by the first author (M.I.), who did not participate in the histopathologic evaluation and was not blinded to clinical outcomes.

### 2.5 Statistical Analysis

Progression‐free interval was defined as the time from surgery to the onset of progressive disease (PD), including recurrence or metastasis, confirmed by diagnostic imaging, cytology or histopathology. Overall survival time was defined as the time from surgery to death from any cause. Dogs lost to follow‐up or still alive were censored at the date of last contact.

PFI and OST were analysed using the Kaplan–Meier method and compared using the log‐rank test. Variables assessed included age, body weight, clinical signs, *HER2* mutation status, HER2 immunoreactivity score, TIL density, the presence of LAs, tumour size classification (CLCSC), lymph node status (N0 vs. N1, N2), surgical margin status, histologic grade and pattern and adjuvant chemotherapy. HER2 immunoreactivity was scored on a scale from 0 to 3, with scores of 0 and 1 classified as negative, and scores of 2 and 3 classified as positive [7, 15]. Lymph node metastasis was evaluated by histopathologic examination, and as in previous reports, for dogs in which lymph node resection or biopsy was not performed, lymph node status was recorded as N0 [2, 5, 7, 16]. Dogs that died perioperatively were excluded from survival analyses of adjuvant chemotherapy. Pairwise correlations among intratumoural CD8+ T cells, CD20+ B cells and Foxp3+ cells were examined using Spearman's correlation test. Cox proportional hazards regression was used to identify variables significantly associated with PFI and OST. Variables with *p* < 0.05 in univariable analysis were included in multivariable models. Fisher's exact test or the Mann–Whitney U test was used to assess associations between the presence of LAs and other variables. Continuous variables are reported as medians and interquartile ranges (IQR). A *p‐*value < 0.05 was considered statistically significant. Statistical analysis was conducted using EZR (Saitama Medical Centre, Jichi Medical University, Saitama, Japan).

## 3 Results

### 3.1 Patient Population

Seventy‐one client‐owned dogs met the inclusion criteria. Clinicopathologic characteristics and postoperative outcomes were largely similar to those reported previously in the same institutional cohort, which evaluated *HER2* mutations in cPACs (Table S1) [7]. *HER2* mutation and IHC data were available for all cases and were obtained as previously described [7]. In brief, *HER2* mutations were detected in 20 dogs (29.0%), and HER2 protein expression was positive (IHC score 2+ or 3+) in 50 dogs (70.4%). Detailed clinicopathologic variables, including age, breed, tumour size classification, lymph node status and histologic grade, are summarised in Table S1. At the time of analysis, 57 dogs (80.3%) had died, seven dogs (9.9%) were alive and seven dogs (9.9%) were lost to follow‐up. Postoperative PD was documented in 33 dogs (46.5%), and no dogs received HER2‐targeted therapy. Median PFI and OST were 754 days (95% CI, 375–not reached) and 716 days (95% CI, 399–936), respectively.

### 3.2 Tumour Microenvironment Profiles

The median densities of intratumoural CD8+, CD20+ and Foxp3+ cells were 130.0 cells/mm^2^ (IQR, 82.3–189.5), 398.3 cells/mm^2^ (IQR, 240.9–581.9) and 79.3 cells/mm^2^ (IQR, 41.8–129.5), respectively. The median CD8/Foxp3 ratio was 1.47 (IQR, 0.99–3.05). Weak but statistically significant positive correlations were observed between CD8+ and CD20+ cell densities (*r* = 0.24, 95% CI 0.004–0.45, *p* = 0.046), between CD8+ and Foxp3+ cell densities (*r* = 0.30, 95% CI 0.07–0.50, *p* = 0.01) and between CD20+ and Foxp3+ cell densities (*r* = 0.27, 95% CI 0.04–0.47, *p* = 0.02) (Figure S1).

**FIGURE 1:**
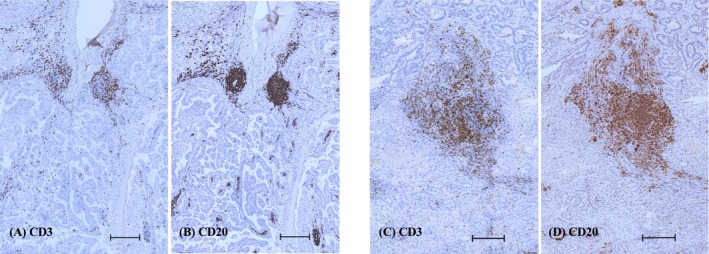
Serial sections from two dogs with surgically resected canine pulmonary adenocarcinomas demonstrating intratumoural lymphoid aggregates (LAs). (A, B) Typical LA from a 10‐year‐old spayed female Toy Poodle. (A) Anti‐CD3 immunohistochemistry (IHC) demonstrating the distribution of T cells. (B) Anti‐CD20 IHC demonstrating the distribution of B cells. (C, D) Large LA from an 11‐year‐old spayed female mixed‐breed dog. (C) Anti‐CD3 IHC demonstrating the distribution of T cells. (D) Anti‐CD20 IHC demonstrating the distribution of B cells. Diaminobenzidine was used as the chromogen, and sections were counterstained with Mayer's haematoxylin. Scale bars = 200 μm.

Representative CD3 and CD20 immunostaining of intratumoural LAs are shown in Figure 1. LAs were identified in 49 dogs (69.0%), and typically consisted of tightly clustered CD20+ B cells with admixed CD3+ T cells. Tumours with LAs showed significantly higher CD20+ cell density (*p* < 0.001) and Foxp3+ cell density (*p* = 0.04) than those without LAs, whereas CD8+ cell density did not differ between groups (Table 1). Lower‐magnification overview images corresponding to Figure 1 are provided in Figures S2 and S3. Representative CD8+, CD20+ and Foxp3+ IHC images are provided in Figure S4.

**TABLE 1:** Comparison of clinical, histological and molecular characteristics according to the presence of intratumoural lymphoid aggregates in dogs with surgically resected pulmonary adenocarcinoma.

Variables	LA absent (*n* = 22)	LA present (*n* = 49)	*p*
Median age, years (IQR)	11.5 (9.0–12.75)	12.0 (10.0–13.0)	0.546
Median body weight, kg (IQR)	5.61 (4.13–9.35)	6.3 (5.1–13.0)	0.723
Clinical signs			1.0
No	10 (14.1%)	21 (29.6%)	
YES	12 (16.9%)	28 (39.4%)	
Madian tumour size, mm (IQR)	50.0 (22.25–63.5)	38.0 (28.9–51.0)	0.452
Lymph node status			0.089
N0	21 (29.6%)	38 (53.5%)	
N1, 2	1 (1.4%)	11 (15.5%)	
Margin			0.711
Complete	20 (28.2%)	42 (59.2%)	
Incomplete	2 (2.8%)	7 (9.9%)	
Adjuvant chemotherapy			0.587
No	15 (22.4%)	29 (43.3%)	
Yes	6 (9.0%)	17 (25.4%)	
Histologic grade			0.184
Grade 1	18 (25.4%)	29 (40.8%)	
Grade 2	4 (5.6%)	18 (25.4%)	
Grade 3	0	2 (2.8%)	
Histologic classification			1.0
Papillary	19 (26.8%)	42 (59.2%)	
Others	3 (4.2%)	7 (9.9%)	
HER2 expression			0.849
1+	7 (9.9%)	14 (19.7%)	
2+	10 (14.1%)	20 (28.2%)	
3+	5 (7.0%)	15 (21.1%)	
*HER2* mutation			0.387
Negative	13 (18.8%)	36 (52.2%)	
Positive	8 (11.6%)	12 (17.4%)	
Median CD8+ cells, mm^2^ (IQR)	120.68 (80.59–168.78)	140.08 (87.76–203.38)	0.526
Median CD20+ cells, mm^2^ (IQR)	263.71 (180.0–380.17)	466.67 (314.77–637.97)	< 0.001
Median Foxp3+ cells, mm^2^ (IQR)	69.20 (19.20–107.59)	94.51 (56.54–151.90)	0.04
CD8/Fxop3 ratio (IQR)	1.85 (1.11–6.85)	1.46 (0.92–2.83)	0.20

### 3.3 Prognostic Analysis

The results of univariable and multivariable analyses are summarised in Table S2, Tables 2 and 3. Univariable findings are presented separately for clinicopathological variables (Table S2) and for HER2 mutation and IHC variables (Table 2). In the clinicopathological set, PFI was associated with clinical signs, tumour size classification (CLCSC), lymph node metastasis, surgical margin status and histological grade, whereas OST was associated with age, clinical signs, tumour size > 7 cm and surgical margin status. These determinants were largely similar to those identified in our previous *HER2* mutation study [7]. Among variables relating to HER2 status and IHC, PFI was associated with HER2 mutation status and the presence of LAs, whereas no association with OST was observed.

**TABLE 2:** Univariable Cox regression for progression‐free interval and overall survival time by human epidermal growth factor receptor 2 mutation status and immunohistochemical variables in dogs with surgically resected pulmonary adenocarcinoma.

Variables	*N*	Progression‐free interval	*p*	Overall survival time	*p*
		Hazard ratio (95% CI)		Hazard ratio (95% CI)	
CD8+ cells	71	1.00 (1.00–1.01)	0.108	1.00 (0.997–1.003)	0.989
CD20+ cells	71	1.00 (0.999–1.002)	0.543	1.00 (0.999–1.001)	0.917
Foxp3+ cells	71	1.00 (0.997–1.01)	0.567	1.00 (0.996–1.004)	0.951
CD8/Foxp3 ratio	71	1.02 (0.960–1.084)	0.522	1.04 (1.001–1.073)	0.043
*HER2* mutation	69				
Negative	49	Reference		Reference	
Positive	20	0.33 (0.13–0.87)	0.025	0.86 (0.47–1.54)	0.605
HER2 expression	71				
Negative	21	Reference		Reference	
Positive	50	0.95 (0.61–1.46)	0.804	0.92 (0.65–1.28)	0.61
Presence of LA	71				
No	22	Reference		Reference	
Yes	49	2.49 (1.03–6.05)	0.043	1.33 (0.75–2.36)	0.33

**TABLE 3:** Multivariable Cox regression analysis of risk factors for the progression‐free interval and overall survival time in dogs with surgically resected pulmonary adenocarcinomas.

Variables	*N*	Progression‐free interval	*p*	Overall survival time	*p*
		Hazard ratio (95% CI)		Hazard ratio (95% CI)	
Age	71	—	—	1.18 (1.04–1.35)	0.013
Clinical signs	71				
No	31	Reference		Reference	
Yes	40	1.02 (0.40–2.60)	0.962	1.63 (0.89–2.97)	0.113
Tumour size classification	71				
≤ 3 cm	24	Reference		Reference	
> 3 to ≤ 5 cm	22	5.71 (1.26–25.98)	0.024	—	—
> 5 to ≤ 7 cm	16	18.14 (3.58–91.78)	< 0.001	—	—
> 7 cm	9	22.80 (2.91–167.60)	0.003	1.75 (0.74–4.16)	0.204
Lymph node status	71				
N0	59	Reference		Reference	
N1, 2	12	1.73 (0.56–5.36)	0.345	2.20 (1.03–4.69)	0.041
Margin	71				
Complete	62	Reference		Reference	
Incomplete	9	5.93 (1.60–21.98)	0.008	1.42 (0.56–3.57)	0.462
Histologic grade	71				
Grade 1	47	Reference			
Grade 2	22	1.44 (0.58–3.57)	0.43	—	—
Grade 3	2	0.29 (0.03–2.92)	0.292	—	—
*HER2* mutation	69				
Negative	49	Reference		—	
Positive	20	0.38 (0.13–1.15)	0.09	—	—
CD8/Foxp3 ratio	71			1.05 (1.07–1.09)	0.004
Presence of LA	71				
No	22	Reference		—	
Yes	49	4.15 (1.15–15.01)	0.03	—	—

**FIGURE 2:**
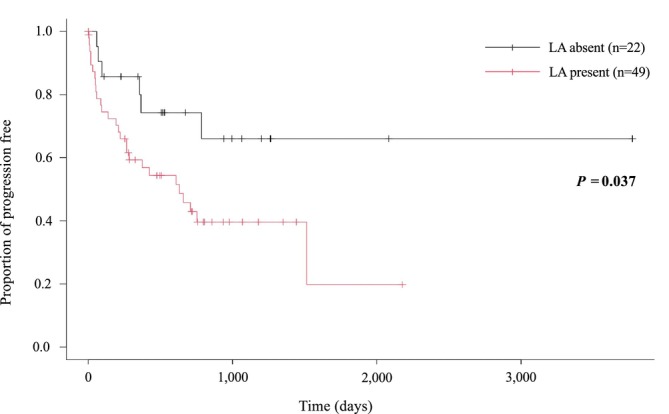
Kaplan–Meier curves of progression‐free interval (PFI) in dogs with surgically resected canine pulmonary adenocarcinomas stratified by the presence of intratumoural lymphoid aggregates (LAs). Dogs with LAs (red line) had significantly shorter PFI than those without LAs (black line). Median PFI was 632 days in the LA‐present group, whereas it was not reached in the LA‐absent group.

In multivariable models (Table 3), tumour size classification (CLCSC), lymph node metastasis, surgical margin status and the presence of LAs were independently associated with shorter PFI; for OST, age, lymph node metastasis and CD8/Foxp3 ratio were independent prognostic factors. Dogs with LAs had a median PFI of 632 days, whereas the median PFI was not reached in dogs without LAs (Figure 2).

## 4 Discussion

We found that intratumoural LAs are common in cPACs and that their presence was independently associated with shorter PFI after surgical resection. This association persisted after adjustment for established clinicopathological determinants, including CLCSC tumour size category, lymph node status and surgical margin status. These findings indicate that immune architecture within the tumour compartment carries prognostic information that is not captured by conventional factors. Although LA‐positive tumours tended to show enrichment of several adverse clinicopathological features, including lymph node metastasis, incomplete surgical margins and higher histologic grade, these differences were not statistically significant in the present cohort. Nevertheless, the association between LA presence and shorter PFI persisted after multivariable adjustment, although residual confounding cannot be fully excluded given the retrospective nature of this study.

In our cohort, the presence of LAs was associated with a shorter PFI, whereas many human NSCLC studies have linked TLSs with better outcomes9‐11. A likely explanation is that the structures we labelled as lymphoid aggregates may not have been mature TLSs. We defined aggregates by visual review on CD3 and CD20 sections and did not apply NanoString‐based gene‐expression or other transcriptomic assays, and our scoring was visual rather than software‐assisted13. In dogs, reliable IHC for follicular dendritic cell markers and for high endothelial venules is difficult because of the lack of antibodies against canine‐specific molecules, so we could not confirm these components or assign a maturation stage. These methodological and technical constraints make it likely that our aggregates included immature or functionally different structures within tumour nests. This scenario aligns with human immuno‐oncology reviews that describe immature TLSs as unlikely to induce effective antitumour responses and as linked to an immunosuppressed TME and T‐cell dysfunction [23].

cPACs with LAs showed higher intratumoural Foxp3+ and CD20+ cell densities. Enrichment of regulatory T cells can suppress local cytotoxic activity and antigen presentation within tumour nests and may contribute to T‐cell dysfunction. Such suppression may permit microscopic residual disease to expand after resection and bring forward the time to progression, consistent with studies in resected NSCLC showing that higher intratumoural Foxp3+ T‐cell density is associated with increased recurrence risk and shorter recurrence‐free survival [24, 25]. This pattern is also consistent with veterinary data that associate higher intratumoural Foxp3+ counts with adverse outcomes in malignant tumours, including PAC [14]. In addition, the increased CD20+ cell density observed in LA‐positive tumours suggests that these aggregates may not simply reflect productive antitumour humoral immunity. Rather, together with the concurrent increase in Foxp3+ cells and the shorter PFI observed in dogs with LAs, these findings raise the possibility that LAs in cPACs may be associated with an immunosuppressive lymphoid microenvironment. This interpretation is broadly consistent with a recent study of gastric‐type endocervical adenocarcinoma, in which TLS‐positive tumours were associated with poor clinical outcomes and showed enrichment of B cells, PD1 − CD8+ T cells and regulatory T cells; in that study, TLSs were interpreted as being associated with immunosuppression rather than antitumour immune activation [26]. Although we did not phenotype B‐cell subsets, the coexistence of increased CD20+ and Foxp3+ cells in LA‐positive tumours raises the possibility that at least a subset of intratumoural B cells may have functioned in an immunoregulatory manner, potentially analogous to regulatory B cells, rather than supporting effective tumour control.

Another notable finding was that the CD8/Foxp3 ratio was independently associated with OST, whereas CD8+ cell density alone was not. This suggests that the prognostic impact of intratumoural lymphocyte infiltration in cPACs may depend less on the absolute number of CD8+ cells than on the balance between cytotoxic and regulatory immune components. Previous work in stage IA lung adenocarcinoma likewise showed that combined evaluation of CD8+ and FoxP3+ TILs was more informative than assessment of either subset alone, with a CD8‐Low/FoxP3‐High profile identifying patients with particularly poor prognosis [27]. Although our results differed in that a higher CD8/Foxp3 ratio was associated with shorter OST, both studies support the notion that the biological significance of CD8+ infiltration cannot be interpreted independently of the accompanying immunoregulatory context. One possible explanation is that a proportion of intratumoural CD8+ T cells in cPACs may have been quantitatively preserved but functionally ineffective, for example, as exhaustion‐prone or non‐tumour‐reactive bystander populations. Thus, in cPACs, a relatively high CD8/Foxp3 ratio may not necessarily indicate intact antitumour immunity, but may instead reflect an immune infiltrate in which CD8+ cells are present numerically but functionally constrained.

This study has limitations. First, the retrospective design introduces potential selection bias. Second, the long enrolment period spans advances in surgical technique and the introduction of targeted drugs such as toceranib, which may have influenced outcomes. Third, the absence of a standardised follow‐up protocol may have affected consistency in progression and survival assessment. Fourth, in 22 dogs for whom lymph nodes were not resected, lymph node status was recorded as N0. Consequently, histological assessment was not available for all dogs and the impact of nodal metastasis may have been underestimated. Fifth, long‐term storage of formalin‐fixed paraffin‐embedded material may have affected IHC staining quality. Sixth, univariable analyses were not adjusted for multiple comparisons, which increases the risk of Type I error. Seventh, we also attempted IHC assessment of PD‐1 expression; however, the staining quality was insufficient for reliable interpretation. Therefore, immune‐checkpoint status could not be evaluated, and the functional exhaustion status of intratumoural T cells could not be directly determined.

In surgically resected cPACs, LA assessment provides TME‐related information that complements clinicopathological variables in postoperative risk stratification. Although LA presence was associated with earlier postoperative progression in the present cohort, its predictive significance may differ in the context of other treatment modalities, including immunotherapy or radiotherapy, and this should be evaluated in future studies. When combined with CD8, CD20 and Foxp3 counts, as well as the CD8/Foxp3 ratio, LA status may help inform future studies of adjuvant or immune‐modulating therapies.

## Author Contributions

Conceptualisation: Masanao Ichimata, Takuya Mizuno. Data curation: Masanao Ichimata, Yumiko Kagawa, Atsushi Toshima. Writing – original draft: Masanao Ichimata, Takuya Mizuno. Writing – review and editing: All authors. Supervision: Masaya Igase, Takuya Mizuno.

## Funding

The authors have nothing to report.

## Ethics Statement

All owners signed informed‐consent forms to use each formalin‐fixed and paraffin‐embedded tissue specimen.

## Conflicts of Interest

The authors declare no conflicts of interest.

## Supporting information


**Figure S1:** Correlations among CD8+, Foxp3+ and CD20+ cell densities in dogs with surgically resected pulmonary adenocarcinoma.
**Figure S2:**. Low‐magnification immunohistochemical images (×2.5) from the 10‐year‐old spayed female Toy Poodle shown in Figure 1. (A) CD3, (B) CD8, (C) CD20 and (D) Foxp3 immunohistochemistry. Diaminobenzidine was used as the chromogen, and sections were counterstained with Mayer's haematoxylin. Scale bar = 200 μm.
**Figure S3:**. Low‐magnification immunohistochemical images (×2.5) from the 11‐year‐old spayed female mixed‐breed dog shown in Figure 1. (A) CD3, (B) CD8, (C) CD20 and (D) Foxp3 immunohistochemistry. Diaminobenzidine was used as the chromogen, and sections were counterstained with Mayer's haematoxylin. Scale bar = 200 μm.
**Figure S4:**. Representative immunohistochemical images of intratumoural CD8+, CD20+ and Foxp3+ cells in canine pulmonary adenocarcinoma from a 10‐year‐old neutered male Toy Poodle. (A) CD8, (B) CD20 and (C) Foxp3. All panels are from the same case. Diaminobenzidine was used as the chromogen, and sections were counterstained with Mayer's haematoxylin. Scale bar = 200 μm.

**Table S1:** Clinicopathological characteristics of all dogs with surgically resected pulmonary adenocarcinomas.

**Table S2:** Univariable Cox regression analysis of clinicopathological risk factors for the progression‐free interval and overall survival time in dogs with surgically resected pulmonary adenocarcinomas.

## Data Availability

The datasets generated and analysed during the current study are based entirely original research and statistical analyses conducted by the authors. These datasets are available from the corresponding author upon reasonable request and may be used for academic and research purposes. All referenced materials are cited appropriately in the Reference section.
