# Supplementary material for: Prognostic Significance of the Tumour Microenvironment in Surgically Resected Canine Pulmonary Adenocarcinomas
Source: Vet Comp Oncol. 2026 Apr 27;24(3):545–53. doi: 10.1111/vco.70075 (PMC13456590; doi:10.1111/vco.70075)

Supplementary Figure S1

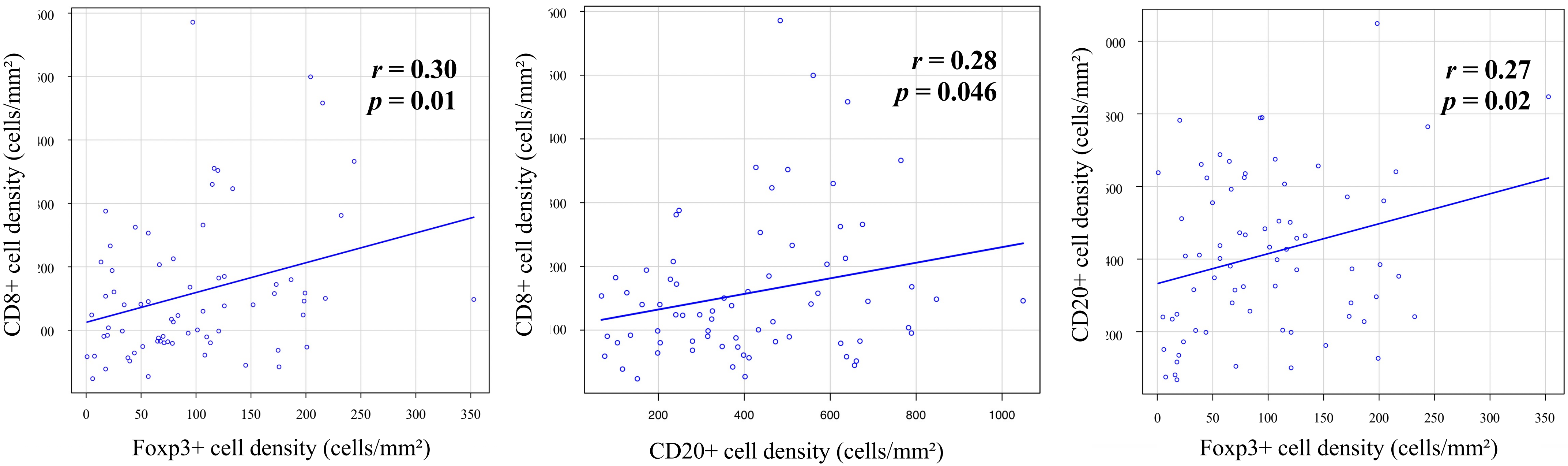

Supplementary Figure S2

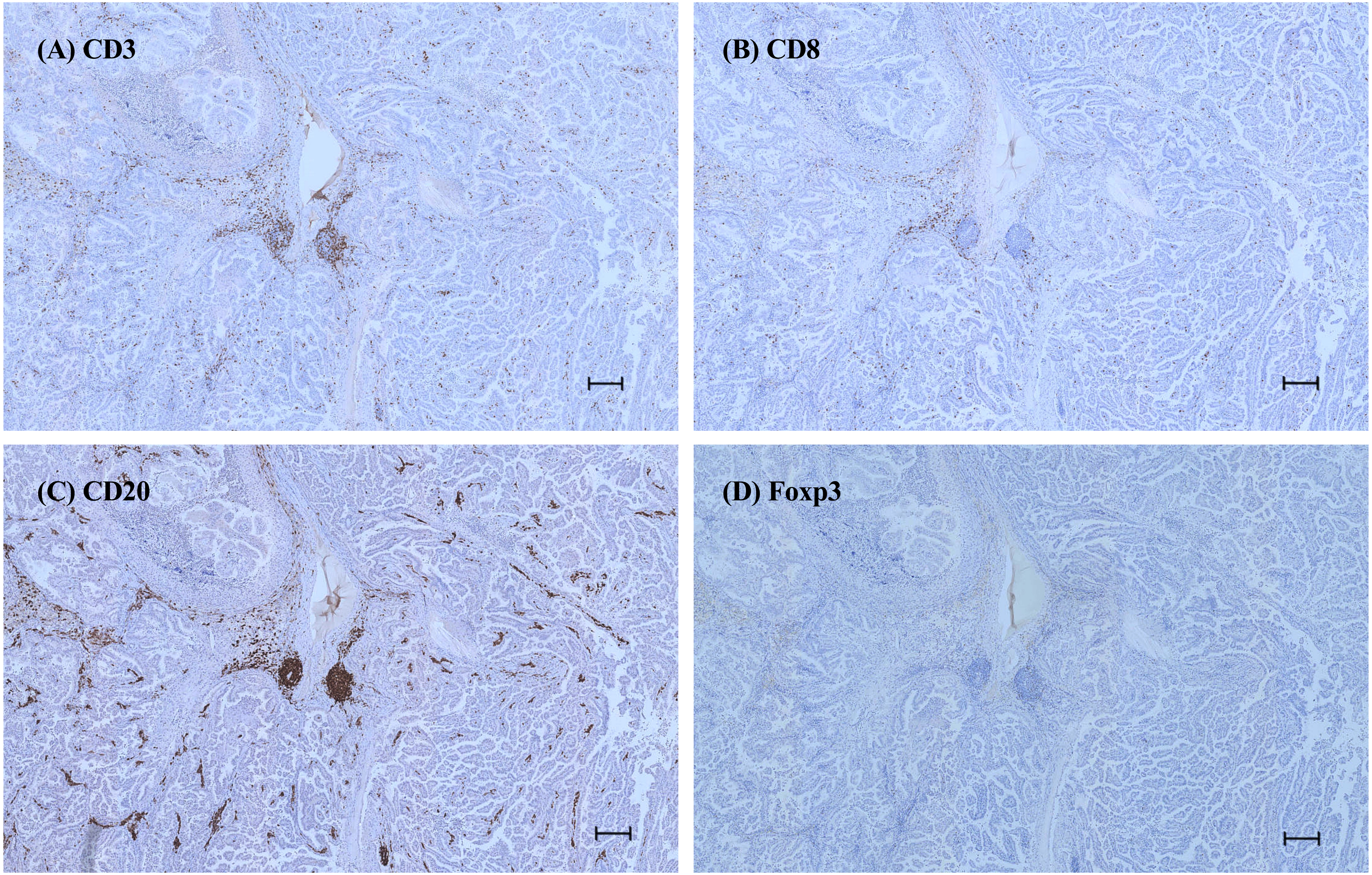

Supplementary Figure S3

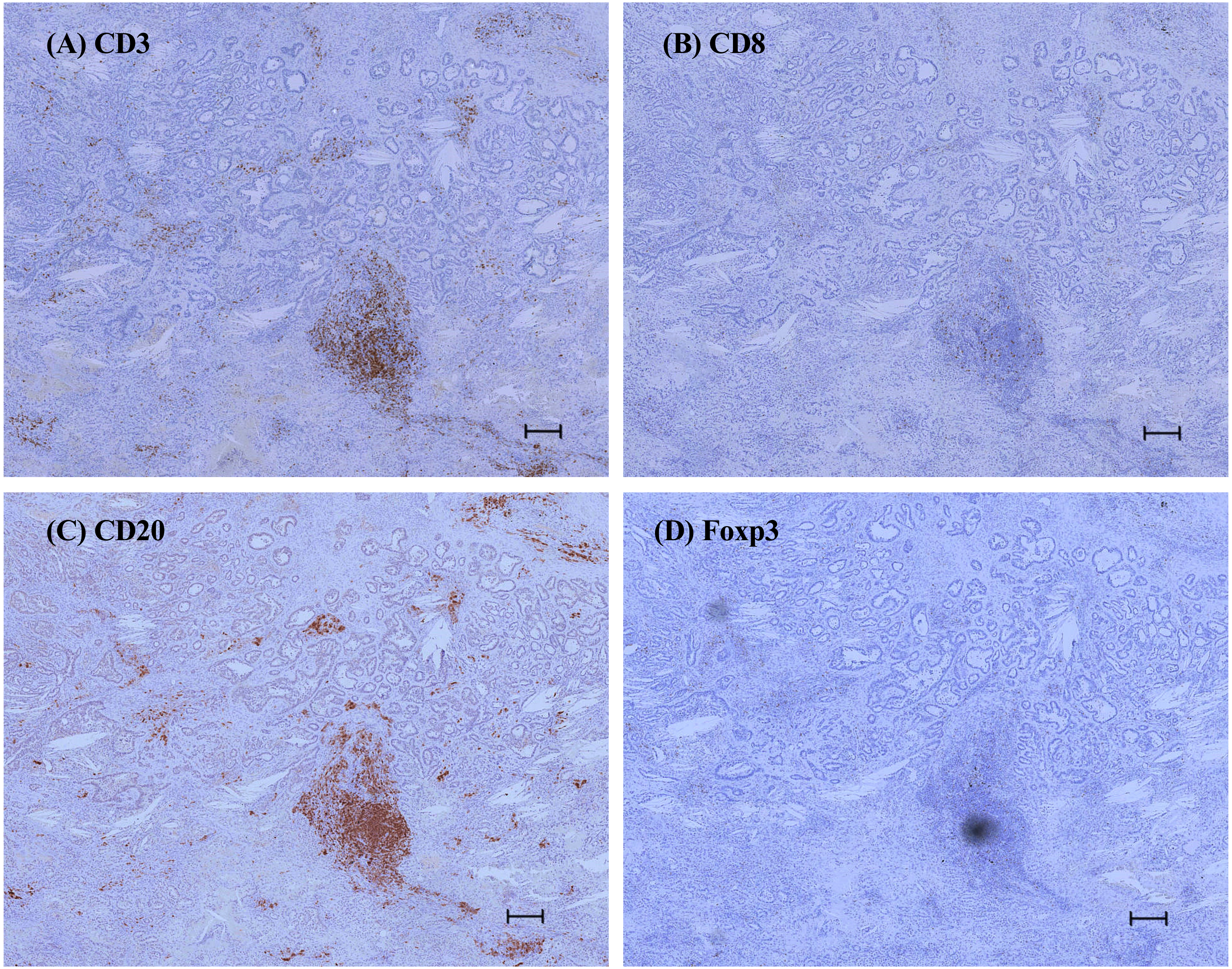

Supplementary Figure S4

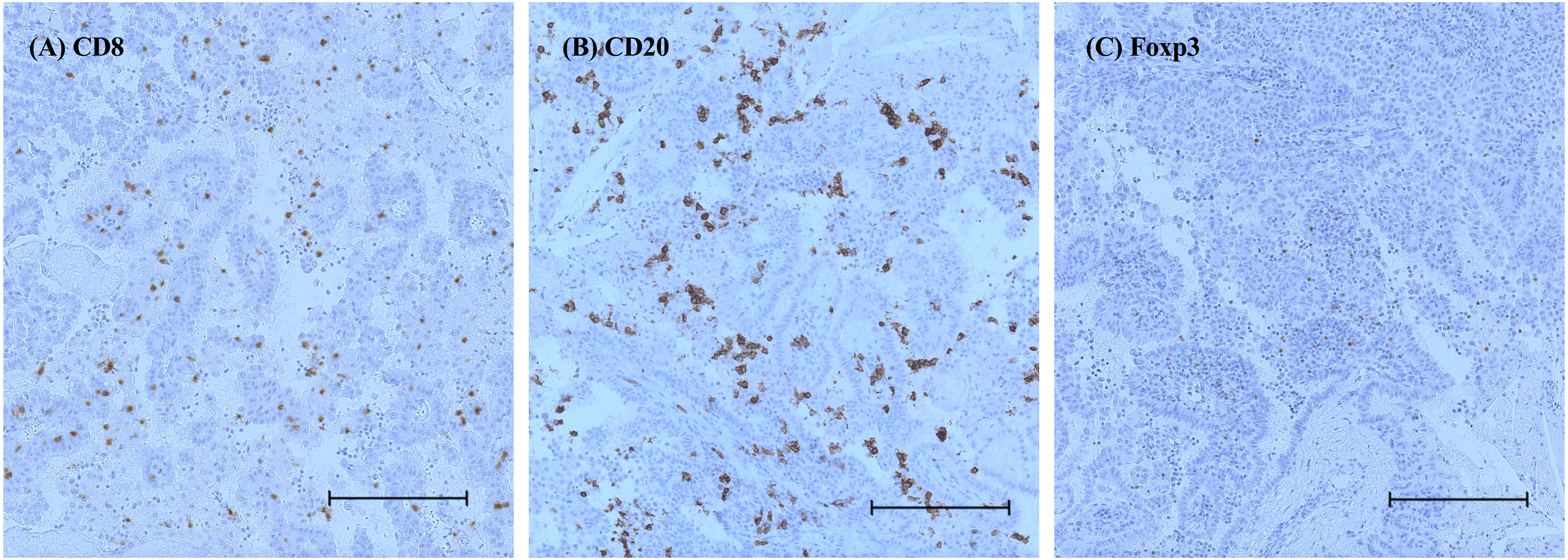

Supplement: Supplementary file 1 [file VCO-24-545-s002.pdf]
