# Supplementary material for: Prognostic Significance of the Tumour Microenvironment in Surgically Resected Canine Pulmonary Adenocarcinomas
Source: Vet Comp Oncol. 2026 Apr 27;24(3):545–53. doi: 10.1111/vco.70075 (PMC13456590; doi:10.1111/vco.70075)
Supplement: Supplementary file 2 [file VCO-24-545-s001.docx]

| **Variables** | **Dogs (n=71)** |
| --- | --- |
| **Median age, years (IQR)** | 12.0 (9.5-13.0) |
| **Median body weight, kg (IQR)** | 6.2 (5.0-9.3) |
| **Clinical signs** |  |
| No | 31 (43.7%) |
| YES | 40 (56.3%) |
| **Median tumor size, mm (IQR)** | 39.0 (26.0-54.0) |
| **Tumor size classification** |  |
| ≤ 3cm | 24 (33.8%) |
| >3 cm to ≤5 cm | 22 (31.0%) |
| >5 cm to ≤7 cm | 16 (22.5%) |
| >7 cm | 9 (12.7%) |
| **Primary tumour location** |  |
| Left cranial lung lobe | 19 (26.8%) |
| Left caudal lung lobe | 15 (21.1%) |
| Right cranial lung lobe | 12 (16.9%) |
| Right middle lung lobe | 6 (8.5%) |
| Right caudal lung lobe | 16 (22.5%) |
| Accessory lung lobe | 3 (4.2%) |
| **Lymph node status** |  |
| N0 | 12 (16.9%) |
| N1, 2 | 59 (83.1%) |
| **Margin** |  |
| Complete | 62 (28.2%) |
| Incomplete | 9 (2.8%) |
| **CLCSC Staging** |  |
| Stage 1 | 19 (26.8%) |
| Stage 2 | 36 (50.7%) |
| Stage 3 | 16 (22.5%) |
| Stage 4 | 0 |
| **Histologic grade** |  |
| Grade 1 | 47 (66.2%) |
| Grade 2 | 22 (31.0%) |
| Grade 3 | 2 (2.8%) |
| **Histologic classification** |  |
| Papillary | 61 (85.9%) |
| Others | 10 (14.1%) |
| **Vascular invasion** |  |
| No | 61 (85.9%) |
| Yes | 10 (14.1%) |
| **Adjuvant chemotherapy** |  |
| No | 44 (65.7%) |
| Yes | 23 (34.3%) |

Supplementary TABLE S1. Clinicopathological characteristics of all dogs with surgically resected pulmonary adenocarcinomas.
