# Supplementary material for: Prognostic Significance of the Tumour Microenvironment in Surgically Resected Canine Pulmonary Adenocarcinomas
Source: Vet Comp Oncol. 2026 Apr 27;24(3):545–53. doi: 10.1111/vco.70075 (PMC13456590; doi:10.1111/vco.70075)
Supplement: Supplementary file 3 [file VCO-24-545-s003.docx]

Supplementary TABLE S2. Univariable Cox regression analysis of clinicopathological risk factors for the progression-free interval and overall survival time in dogs with surgically resected pulmonary adenocarcinomas.

| **Variable** | N | **Progression-free interval** | *P*-value | **Overall survival time** | *P*-value |
| --- | --- | --- | --- | --- | --- |
|  |  | Hazard ratio  (95% confidence interval) |  | Hazard ratio  (95% confidence interval) |  |
| **Age** | 71 | 1.03 (0.87-1.21) | 0.74 | **1.19 (1.0-1.36)** | **0.01** |
| **Body Weight** | 71 | 1.00 (0.96-1.05) | 0.85 | 1.00 (0.97-1.03) | 0.94 |
| **Clinical signs** | 71 |  |  |  |  |
| No | 31 | Reference |  | Reference |  |
| YES | 40 | **2.54 (1.21–5.27)** | **0.01** | **2.00 (1.15-3.49)** | **0.02** |
| **Tumor size classification** | 71 |  | **<0.001** |  | **0.02** |
| ≤3 cm | 24 | Reference |  | Reference |  |
| >3 cm to ≤5 cm | 22 | **7.55 (1.69-33.75)** | **0.01** | 1.10 (0.55-2.21) | 0.79 |
| >5 cm to ≤7 cm | 16 | **15.59 (3.45-70.51)** | **<0.001** | 1.91 (0.92-3.95) | 0.08 |
| >7 cm | 9 | **21.89 (4.47-107.30)** | **<0.001** | **2.89 (1.27-6.60)** | **0.01** |
| **Lymph node status** | 71 |  |  |  |  |
| N0 | 59 | Reference |  | Reference |  |
| N1, 2 | 12 | **4.85 (2.18-10.79)** | **0.001** | **2.40 (1.22-4.70)** | **0.01** |
| **Margin** | 71 |  |  |  |  |
| Complete | 62 | Reference |  | Reference |  |
| Incomplete | 9 | **12.93 (5.20-32.19)** | **<0.001** | **3.44 (1.64-7.20)** | **0.001** |
| **Histologic grade** | 71 |  | **0.003** |  | 0.05 |
| Grade 1 | 47 | Reference |  | Reference |  |
| Grade 2 | 22 | **2.24 (1.09-4.59)** | **0.03** | 1.62 (0.92-2.84) | 0.10 |
| Grade 3 | 2 | **6.45 (1.45-28.74)** | **0.01** | 2.33 (0.55-9.83) | 0.25 |
| **Histologic classification** | 71 |  |  |  |  |
| Papillary | 61 | Reference |  | Reference |  |
| Others | 10 | 0.63 (0.22-1.81) | 0.39 | 0.46 (0.21-1.02) | 0.05 |
| **Adjuvant chemotherapy** | 67 |  |  |  |  |
| No | 44 | Reference |  | Reference |  |
| Yes | 23 | 1.36 (0.67-2.75) | 0.39 | 0.89 (0.50-1.57) | 0.69 |
